# Supplementary material for: A Human Stem Cell-Derived Brain-Liver Chip for Assessing Blood-Brain-Barrier Permeation of Pharmaceutical Drugs
Source: Cells. 2022 Oct 19;11(20):3295. doi: 10.3390/cells11203295 (PMC9600760; doi:10.3390/cells11203295)
Supplement: Supplementary file 1 [file cells-11-03295-s001.zip › cells-1941199-supplementary.pdf]

Supplemental data items

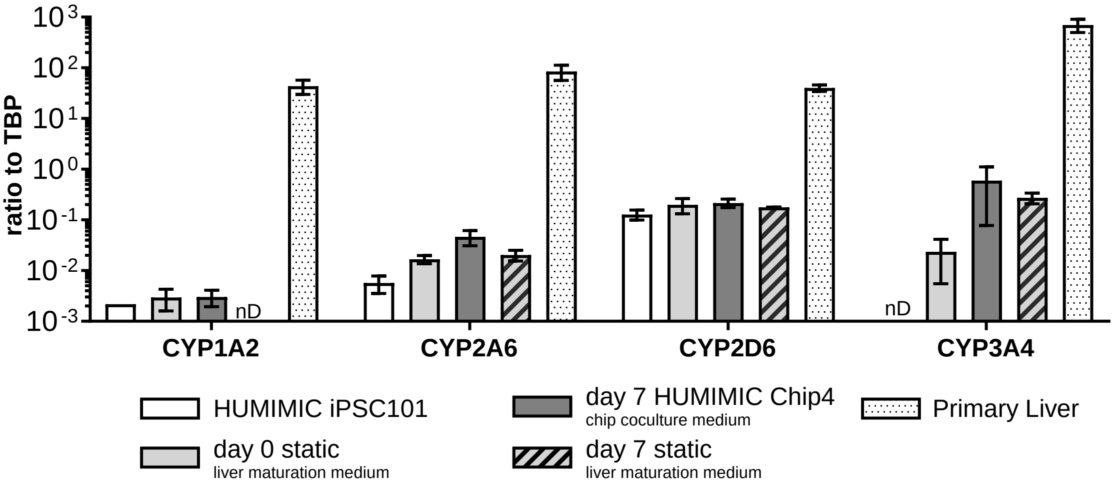

Figure S1: Transcript expression of cytochrom-P450 genes in iPSC-derived liver spheroids, related to Figure 3B. CYP1A2, CYP2A6, CYP2D6 and CYP3A4 expression after formation (day 0) and after 7 days of Chip4 culture in the chip coculture medium and after 7 days of static culture in liver maturation medium. Data are expressed as mean ± s.e.m of three independent experiments (N=3). On the y-axis, the gene expression levels are shown as ratio to the housekeeper gene TBP. HUMIMIC hiPSC101 samples from three different passages and primary liver samples from three donors were used as controls.

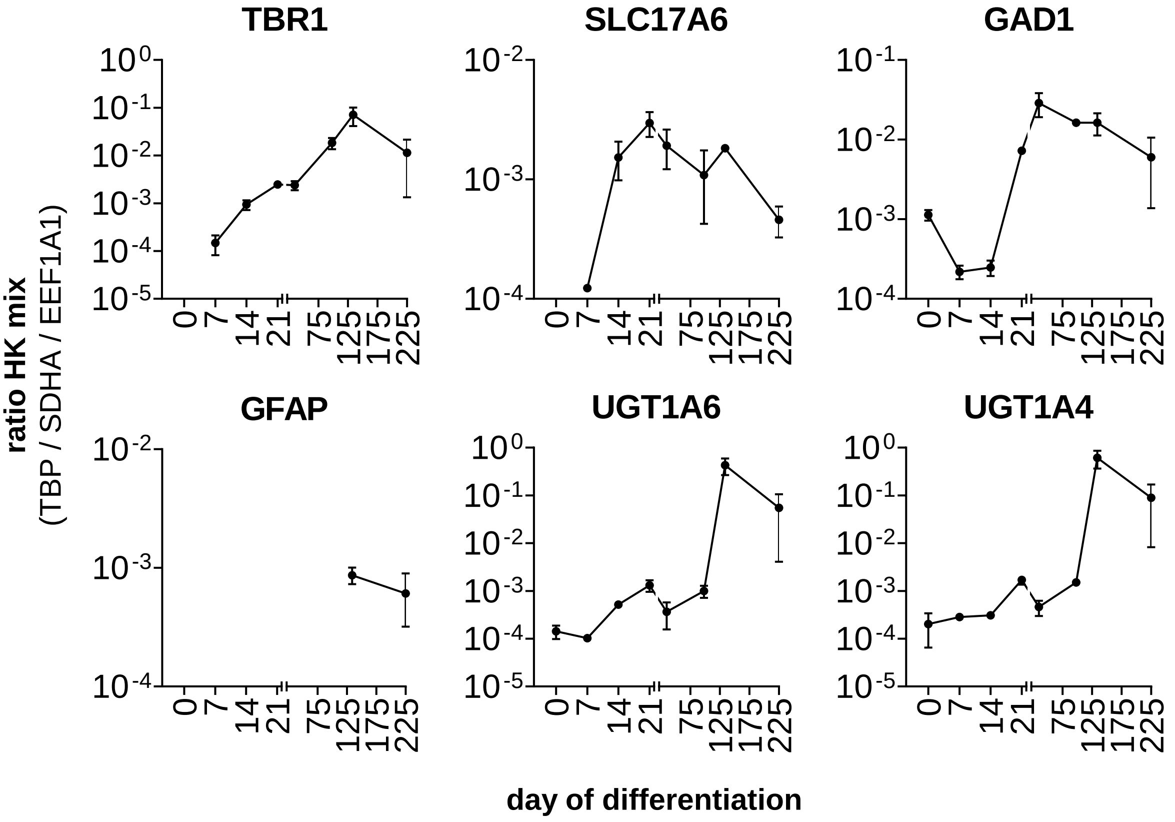

Figure S2: Transcript expression of astrocyte associated genes in neural spheroids in static single culture over time, related to Figure 4E-G. Cortical developmental marker TBR1, glutamatergic neuron marker SLC17A6, GABAergic marker GAD1, astrocyte marker GFAP and UDP-glucuronosyltransferase 1A6

and 1A4 in neural spheroids at day 0, 7, 14 and 21 days of differentiation in the bioreactor and for prolonged cultivation in Erlenmeyer flasks. Data are expressed as mean ± s.e.m from one differentiation run with three to five technical replicates per timepoint (n=3-5). On the y-axis, the gene expression levels in is shown as ratio to the mean expression of the housekeepers TBP, SDHA and EEF1A1.

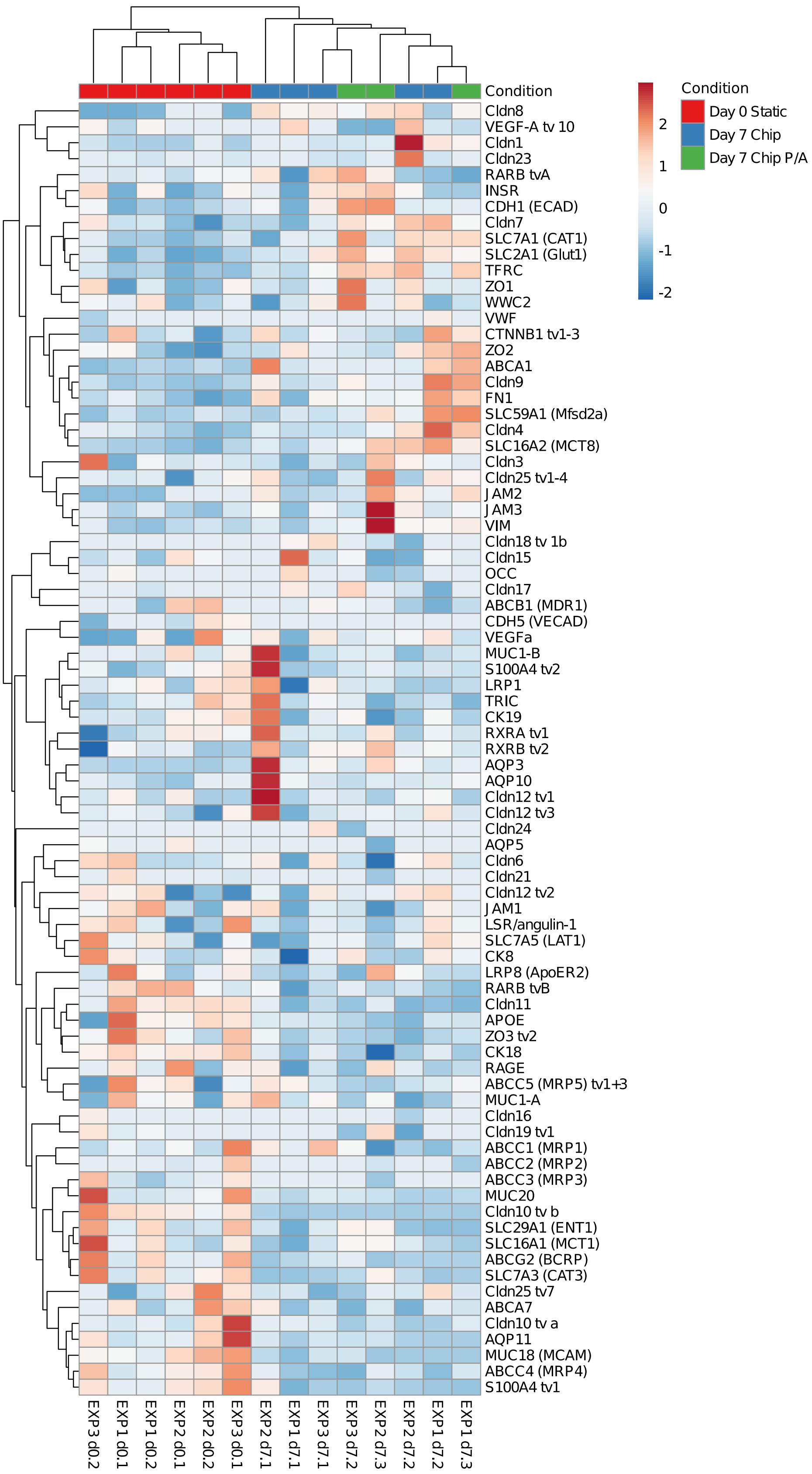

Figure S3: Hierarchical clustering of hiPSC-derived BMEC-like cells before and after Chip4 culture, related to Figure 4D. Heatmap plot of six BMEC-like cell samples from three independent experiment on day 0 (N=3) and eight BMEC-like cell samples from day 7 in the Chip4 from three independent experiments (N=3) – three of the samples were from the atenolol and propranolol treatment group (P/A). Original 2-ΔCT values were ln(x + 1)-transformed. Rows are centered; unit variance scaling is applied

to rows. Imputation is used for missing value estimation. Both rows and columns are clustered using correlation distance and average linkage.

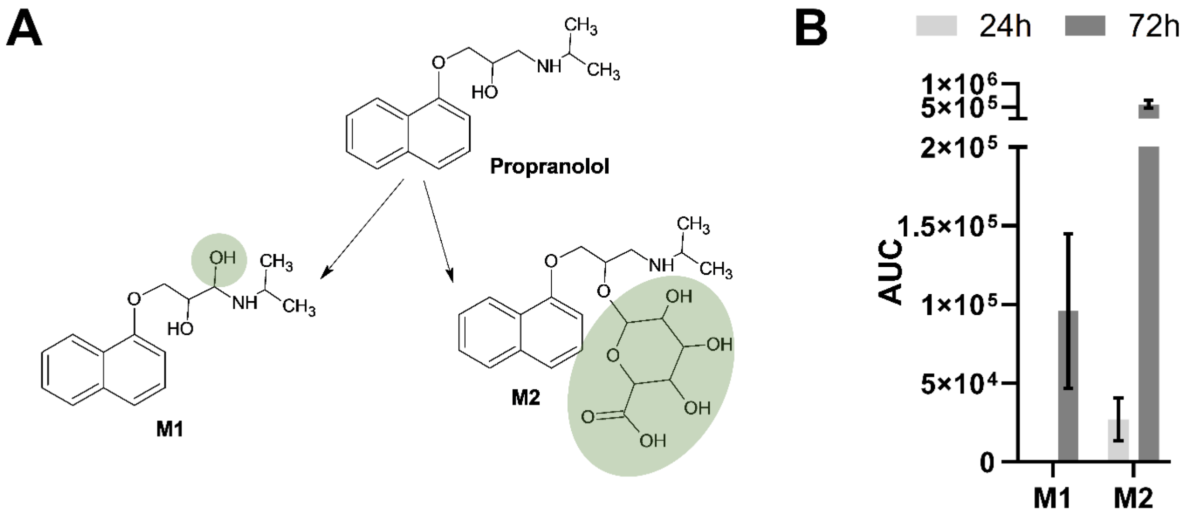

Figure S4: Propranolol metabolization by the liver model under static conditions, related to Figure 3. (**A**) Proposed metabolic pathway for propranolol, metabolite identification is based on accurate mass only. (**B**) Mean AUC of propranolol metabolites + s.e.m. Metabolites were measured in the medium after 24h and 72h of daily of 5 μM propranolol with three technical replicates (n=3).

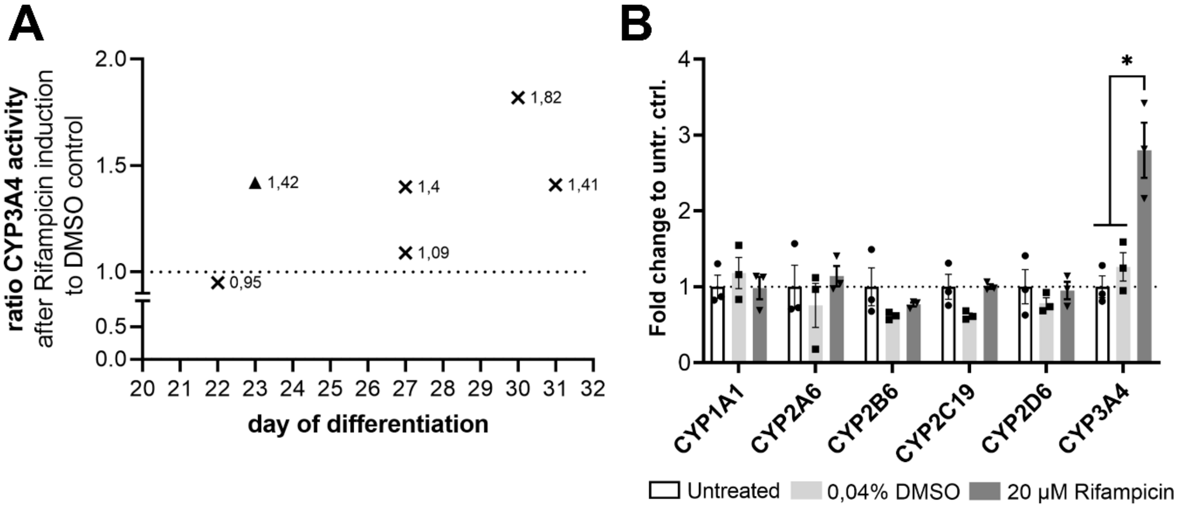

Figure S5: CYP3A4 activity and induction capability in hiPSC-derived liver models, related to Figure 3. (**A**) CYP3A4 activity after 3 days of 20 μM rifampicin application, ratio to DMSO solvent control is shown. Each data point represents one independent differentiation. CYP3A4 activity assay were carried out at different timepoints after start of the hepatic differentiation as indicated by the day of differentiation on the x-axis. (**B**) For one differentiation (marked with a triangle in plot A), mRNA expression levels of selected CYP enzymes were analyzed. Mean fold changes + s.e.m. of the DMSO solvent control and the Rifampicin treated samples to the untreated control of three technical replicates (n=3) are shown. Two-way ANONA with Tukey post-hoc test was used to detect significant upregulations upon rifampicin treatment, *p<0.05.

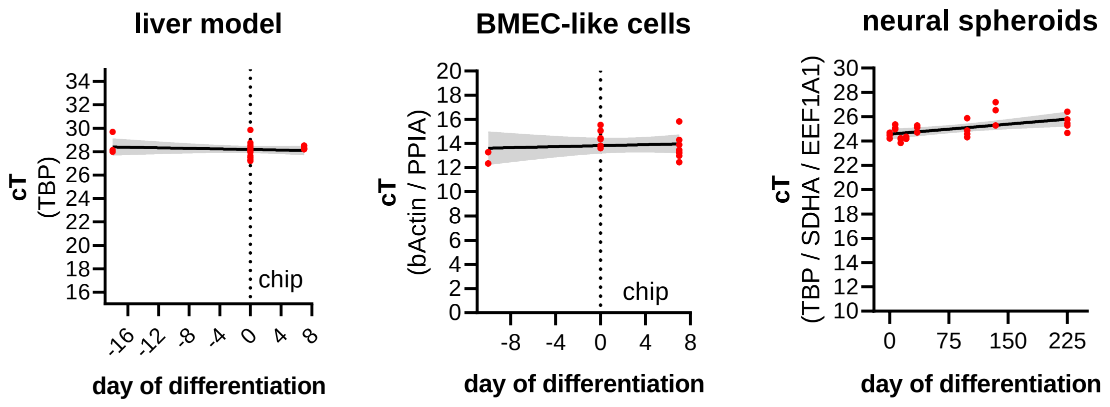

Figure S6: Expression profile of the selected housekeeper genes at different stages of liver, BMEC-like cell and neural spheroid model, related to Figure 3B, 4D and S2. For each housekeeper combination the cT values of the respective day are plotted and linear fits were applied (black lines). The displayed grey areas represent the 95% confidence intervals. For the liver model three iPSC samples from different passages (day-16), liver models before chip culture from 4 experiments with 4 technical replicates each (day 0) and liver models after chip culture from three experiments with 1 – 3 technical replicates each (day 7) are plotted. For the BMEC-like cells two iPSC samples from different passages (day-10), BMEC-like cells before chip culture from 3 experiments with 2 technical replicates each (day 0) and BMEC-like cells after chip culture from three experiments with 2 – 3 technical replicates each (day 7) are plotted. For the neural spheroids three technical replicates from one differentiation at days 0, 7, 14, 21, 35, 98, 134 and 225 of the differentiation are plotted.

Table S1: Medium exchange regimen and volumes in the coculture assay. Medium exchange is marked by "-/+". Media exchanges marked with a "D" for drug indicate that here atenolol and propranolol was applied with the cell culture medium.

timepoint	**Coculture Assay**<br>BBB/brain + liver			
	**Intestine**	**Mix 1**	**Brain**	**Liver**
**volume (inside)**	1000 µL	300 µL	75 µL	300 µL
**volume (below)**			150 µL	
**Total volume in circulation** (+ channels)	1782 µL			
**day 2**	-/+<br>1000 µL	-/+<br>300 µL	/	/
**day 5**	-/+<br>1000 µL **D**	-/+<br>300 µL **D**	-/+<br>75 µL	/
**day 7**	sampling of all media			

Table S2: Table of used primers for liver and neural transcript expression by quantitative RT-PCR Related to Figure 3 B, Figure S1 and Figure S2.

**Gene symbol**	**Gene name**	**NCBI RefSeq**	**Primer sequence**
ABCC2	ATP-binding cassette subfamily C member 2	NM_000392.5	CTGCGGCTCTCATTCAGTCTTTC<br>GCATCCACAGACATCAGGTTCAC
AFP	alpha fetoprotein	NM_001134.3,<br>NM_001354717.2	CTTCCAAACAAAGGCAGCAACAG<br>TCCTGCAGACAATCCAGCAC

ALB	albumin	NM_000477.7	TCAGCTCTGGAAGTCGATGAAAC<br>AGTTGCTCTTTTGTTGCCTTGG
CYP1A2	cytochrome P450 family 1 subfamily A member 2	NM_000761.5	ATCCCCCACAGCACAACAAG<br>CCATGCCAAACAGCATCATC
CYP2A6	cytochrome P450 family 2 subfamily A member 6	NM_000762.6	GTACCCTATGCTGGGCTCTGTG<br>CCTTAGGTGACTGGGAGGACTTG
CYP2D6	cytochrome P450 family 2 subfamily D member 6	NM_000106.6,<br>NM_001025161.3	CTGAAGGATGAGGCCGTCTG<br>CACCGAGAAGCTGAAGTGCTG
CYP3A4	cytochrome P450 family 3 subfamily A member 4	NM_017460.6,<br>NM_001202855.3	GGAAGTGGACCCAGAAACTGC<br>TTACGGTGCCATCCCTTGAC
EEF1A1	eukaryotic translation elongation factor 1 alpha 1	NM_001402.6	GTTGATATGGTTCCTGGCAAGC<br>GCCAGCTCCAGCAGCCTTC
FOXA2	forkhead box A2	NM_021784.5,<br>NM_153675.3	AGGGCTACTCCTCCGTGAG<br>CGACGACATGTTCATGGAGC
GAD1	glutamate decarboxylase 1	NM_000817.3	TGAAGCCAAACAGAAGGGATATG<br>AGTTTATGGCGGTGCTTCCTG
GFAP	glial fibrillary acidic protein	NM_002055.5,<br>NM_001363846.2,<br>NM_001242376.3,<br>NM_001131019.3	GGAACAGCAAAACAAGGCGCT<br>GTGGCTTCATCTGCTTCCTGTCT
HNF4a	hepatocyte nuclear factor 4 alpha	NM_000457.6,<br>NM_001287183.2,<br>NM_175914.5,<br>NM_178849.3,<br>NM_001030004.3,<br>NM_001030003.3,<br>NM_001258355.2,<br>NM_001287184.2,<br>NM_178850.3,<br>NM_001287182.2	ATACGCATCCTTGACGAGCTG<br>CTGGCGGTCGTTGATGTAGTC
SDHA	succinate dehydrogenase complex flavoprotein subunit A	NM_004168.4,<br>NM_001294332.2,<br>NM_001330758.2	TCCAGGGGCAACAGAAGAAG<br>TTGTCTCATCAGTAGGAGCGAATG
SLC17A6	solute carrier family 17 member 6	NM_020346.3	GCAAGGTCATCAAGGAGAAAGC<br>CAGCTCCGAAAACCCTGTTG
TBP	TATA-Box Binding Protein	NM_003194.5,<br>NM_001172085.2	CCTTGTGCTCACCCACCAAC<br>TCGTCTTCCTGAATCCCTTTAGAATAG
TBR1	T-box brain transcription factor 1	NM_006593.4	AGGGAAGGCGCATGTTTCCTTT<br>TGCACATTGGTGTCCGCTTTG
UGT1A4	UDP glucuronosyltransferase family 1 member A4	NM_007120.3	TTACGCTGGGCTACACTCAAGG<br>ACAACACCTATGAAGGGCCAAAG
UGT1A6	UDP glucuronosyltransferase family 1 member A6	NM_001072.4	CTGCCTCCTTCGCTCATTTC<br>CTTCAGGCACCACCACTACAATC

Table S3: Details of used antibodies for immunocytochemistry
Related to Figure 3 C and to Figure 4 C, E and F.

Antigen		Clone	Host	Isotype	Manufacturer	Cat. Nr.	Dil.
Albumin	ALB	HSA-11	mouse	IgG2a	Sigma-Aldrich	A6684	1:100
Claudin 5	claudin 5	polyclonal	rabbit	IgG	Abcam	ab15106	1:100
Glial fibrillary acidic protein	GFAP	polyclonal	rabbit	IgG	Sigma-Aldrich	G4546	1:100
Glucose transporter 1 / SLC2A1	GLUT1	SPM498	mouse	IgG2a	Novus Biologicals	NBP1-35926	1:100
Hepatocyte nuclear factor 4 alpha	HNF4a	F.674.9	rabbit	IgG	Thermo Fisher	MA5-14891	1:50
Ki-67	ki67	20Raj1	mouse	IgG1	Thermo Fisher	14-5699-82	1:100
Beta-3 Tubulin	TUBB3	2G10 TB3	mouse	IgG2a	Thermo Fisher	14-4510 82	1:100
Vascular endothelial cadherin	VE-Cad	F-8	mouse	IgG1	Santa Cruz	sc-9989	1:100
Tight junction protein 1	ZO-1	polyclonal	rabbit	IgG	Proteintech	21773-1-AP	1:200
Microtubule-associated protein 2	MAP2	AP20	mouse	IgG1	Sigma-Aldrich	MAB3418	1:100
Synaptophysin 1	SYP	polyclonal	rabbit	IgG	SynapticSystems	101 002	1:100
Mouse IgG-CF594		polyclonal	Goat	IgG	Biotium	20119	1:200
Rabbit IgG-CF488A		polyclonal	Goat	IgG	Biotium	20019	1:200

Table S4: Commercial kits used for analysis at the Indiko clinical chemistry analyzer

Product	Manufacturer	Cat. Nr.
Albumin in Urine/CSF FS (Microalbumin) Kit	DiaSys	10242
Fluitest Lactate Kit	Analyticon Biotechnologies	3011
Glucose (HK) Kit	Thermo Fisher Diagnostics	981779
Lactate dehydrogenase (LDH) IFCC	Thermo Fisher Diagnostics	981782

Supplemental Experimental Procedures

Immunofluorescence staining

For all immunohistochemistry staining, a negative control staining of the same tissue without the primary antibody were prepared to test the tissue for unspecific binding of the secondary antibody. Exposure settings were adjusted so that no considerable signal was detected in the negative control staining.

BMECs on cell culture insert membranes were washed with $PBS^{-/-}$. The cells were then fixed with 4% PFA (Thermo Fisher) for 10 min at room temperature and afterwards stored in $PBS^{-/-}$ at 4 °C until staining. The cells on the membrane or in the cell culture plate were permeabilized with 100 µL 0.2% Triton-X100 (VWR Chemicals) in $PBS^{-/-}$ (perm buffer) for 5 min at room temperature. The perm buffer was removed and the membranes incubated in $PBS^{-/-}$ + 5% goat serum (Meridian Life Sciences) + 0.02% saponin (Sigma-Aldrich) + 0.1% Triton-X100 (block buffer) for 20 min at room temperature. The block buffer was removed and the membranes incubated with the primary antibodies diluted in block buffer overnight in the dark at 4°C. The next day the staining solution was removed and the cells were washed three times for 5 min with 0.05% Tween-20 (Atlas Chemicals) in $PBS^{-/-}$ (wash buffer). Cells were incubated with secondary antibodies and 1 µg/mL DAPI (Sigma-Aldrich) in $PBS^{-/-}$ for one hour in the dark at room temperature. Alternatively, membranes were incubated with 3 U/L Phalloidin-DyLight 488 (Thmero Fisher) to stain actin filaments. Afterwards, the membranes were washed again three times for 5 min with wash buffer. Primary and secondary antibodies were diluted as indicated in **Table S3**.

Liver and neural spheroids were used for preparation and staining of cryostat micro-sections. Liver spheroids were washed with $PBS^{-/-}$ to remove cell culture medium, transferred without excess liquid into Tissue-Tek O.C.T. compound (Sakura Finetek), and directly frozen at -80 °C. Because of their large size, neural spheroids were first dehydrated with sucrose before they were embedded. Neural spheroids were washed with $PBS^{-/-}$ to remove remaining traces of cell culture medium first, then fixated with 4% PFA for 1 h at room temperature, and afterwards stored in 15% w/v sucrose solution (Sigma-Aldrich) overnight at 4 °C. The next day the spheroids were transferred into 30% w/v sucrose solution and incubated for 4 to 6 h at room temperature. The spheroids were then transferred without excess liquid into Tissue-Tek O.C.T. compound and frozen at -80 °C. A cryostat microtome (CryoStar NX70, Thermo Fisher) was used to prepare micro-tissue sections of 8 µm thickness. Multiple sections were collected on adhesion slides and stored at -20 °C until staining.

Directly before staining, the cryosections were fixated and permeabilized in ice-cold acetone (VWR Chemicals) at -20 °C for 10 min. Slides were then washed twice in $PBS^{+/+}$ for 5 min at room temperature. The slides were then briefly allowed to dry and the individual tissue sections encircled with a liquid-blocking pen (Science Services). The primary antibodies were diluted in 10 % goat serum in $PBS^{-/-}$ and applied with a volume of 35 µL to the encircled sections. Incubation with the primary antibodies was done overnight at 4 °C in the dark. The next day, the slides were washed again three times in $PBS^{+/+}$ for 5 min at room temperature. The encircled sections were incubated with secondary antibodies and 1 µg/mL DAPI in $PBS^{-/-}$ for one hour in the dark at room temperature and afterwards washed again three times for 5 min with $PBS^{+/+}$. Finally, sections were embedded in mounting solution (Imsol Mount, VWR), covered with a cover slip and left for drying in the dark at room temperature until the next day. Primary and secondary antibodies were diluted as indicated in **Table S3**.

TUNEL staining

Detection of apoptotic cells was done by labeling of DNA fragmentation with the terminal deoxynucleotidyl transferase dUTP nick end labeling (TUNEL) method. The Apo-Direct Apoptosis Detection Kit (Thermo Fisher) was used according to the manufacturer protocol.

Cryosections on glass slides were fixated and permeabilized in ice-cold acetone at -20 °C for 10 min. Slides were then washed twice with $PBS^{+/+}$ for 5 min at room temperature. The slides were then briefly allowed to dry and the individual tissue sections encircled with a liquid-blocking pen. Positive controls sections were treated with 35 µL of 600 U/mL DNase I diluted in 50 mM Tris-HCl supplemented with 1 mg/mL BSA for 10 min at room temperature. Slides were then washed with wash buffer for 5 min at room temperature and each section subsequently incubated with 35 µL DNA labeling solution for 60 min at 37 °C. The DNA labeling solution was prepared according to the manufacturer's protocol by mixing the TdT reaction buffer, TdT enzyme, fluorescein-dUTP and water. For the negative control, the TdT enzyme was replaced with water. Afterwards DNA labeling solution was replaced by rinse buffer for 5 min and the slides subsequently washed two times for 5 min in $PBS^{-/-}$. To visualize proliferating cells in the same sections, slides were stained for the proliferation marker ki67 diluted as indicated in **Table S3**.

qPCR analysis

RNA was isolated from cell lysates with the NucleoSpin RNA XS Kit (Machery-Nagel) according to the manufacturer's protocol. RNA concentrations were calculated by measurement of absorbance at 260 nm with the CLARIOstar plate reader (BMG Labtech). Transcription into cDNA was performed with 150 ng of the isolated RNA with the TaqMan Reverse Transcription Kit at a QuantStudio LightCycler (Thermo Fisher). The synthesized cDNA was used for gene transcript quantification by quantitative PCR.

For liver and neural cell samples, the reaction was performed in 384 well PCR microplates using the SensiFAST SYBR Lo-ROX Kit (Bioline) according to the manufacturer's protocol. The kit contains a ready-to-use master mix that consists of the SYBR green I dye, AmpliTaq fast DNA polymerase, Uracil-DNA glycosylase, ROX dye passive reference, dNTPs and optimized buffer components. The amplification mix was prepared with 0.5 µL of the cDNA, SensiFAST SYBR Lo-ROX ready-to-use master-mix and 0.25 µM primer as listed in **Table S2**. Finally, the PCR was performed using the QuantStudio 5 Real-Time PCR System (384 well system) at an annealing and elongation temperature of 64°C (30 seconds). Relative expression was quantified by use of the comparative cycle threshold (Ct) method, normalized to TBP expression (liver spheroids) or EEF1A1, SDHA and TBP expression (neural spheroids).

For BMEC-like cells, the samples were sent frozen to the AIT Austrian Institute of Technology GmbH for qPCR analysis on a 96.96 dynamic array IFC (Fluidigm Corporation) according to the manufacturer's protocol. In brief, ninety-four targets were investigated: Claudin-1, Claudin-2 tv3, Claudin-3, Claudin-4, Claudin-5, Claudin-6, Claudin-7, Claudin-8, Claudin-9, Claudin-10 tv a, Claudin-10 tv b, Claudin-11, Claudin-12 tv1, Claudin-12 tv2, Claudin-12 tv3, Claudin-14, Claudin-15, Claudin-16, Claudin-17, Claudin-18 tv 1b, Claudin-18 tv2a, Claudin-19 tv1, Claudin-20, Claudin-22, Claudin-23, Claudin-24, Claudin-21, Jam-1, Jam-2, Jam-3, ZO-1, ZO-2, ZO-3 tv2, VWF, GLUT1, Occludin, CDH5, ABCB1, MRP1, MRP2, MRP3, MRP5 tv1+3, MRP4, BCRP, Tricellulin, CAT1, CAT3, ENT1, Insulin receptor, LAT1, LRP1, LRP8, MCT1, MCT8, Transferrin receptor, VEGF-A, VEGF-A tv 10, LSR, WWC2, CK-8, CK-18, CK-19, AQP3, AQP5, AQP10, MUC 1A, AQP 11, MUC 1B, MUC 18, MUC 20, CDH1, beta-catenin tv1-3, Vimentin, Fibronectin, S100A4 tv1, S100A4 tv2, Claudin-25 tv1-4, Claudin-25 tv7, Claudin-27, Claudin-26 tv3, ABCA1, ABCA7, ApoE, hRAR a tv1-4, hRAR b tvA, hRAR b tvB, hRxR a tv1, hRxR b tv2, Mfsd2a, RAGE tvA, Pecam-1, PPIA, b-actin, B2M, GAPDH. cDNA samples were preamplified using tenfold concentrated primer pools mixing with Qiagen Mastermix applying the following program: 15 min at 95 °C for HotStar Plus Taq Polymerase (Qiagen, Cat. No. 203603), 18 cycles (with 40 s at 95 °C, 40 s at 60 °C, 80 s at 72 °C) and 7 min at 72 °C. High-throughput qPCR was accomplished with a Biomark System containing an IFC Controller HX and 96.96 Dynamic ArraysTM IFC, according to the manufacturer's instructions. In brief, 96 sample wells were loaded with DNA Mix containing Tagman GeneExpression Mastermix, DNA binding dye sample loading reagent, EvaGreen binding dye and 1:8 diluted preamplified cDNA, whereas 96 target wells were filled with the Assay mix containing Assay

loading reagent and according primers. Relative expression was quantified by use of the comparative cycle threshold (Ct) method, normalized to PPIA and beta-actin expression. Fold difference calculation was done by normalization of expression levels to the BMEC-like cell sample group before chip culture.

Hierarchical clustering and of the $2^{-\Delta Ct}$ values were carried out with the online Clustvis tool [1]. Original $2^{-\Delta Ct}$ values were ln(x + 1)-transformed. Rows were centered and unit variance scaling was applied to rows. Imputation was used for missing value estimation. Both rows and columns were clustered using correlation distance and average linkage.

Expression stability of the selected housekeeper genes is shown in **Figure S6** for the liver model, the BMEC-like cells and the neural spheroid model by stable cT values during the differentiation process.

Measurement of glucose and lactate concentration in the cell culture supernatant

Supernatant samples were collected as part of the medium exchange and used for measurement of the glucose, lactate, albumin and lactate dehydrogenase (LDH) concentration with the Indiko clinical chemistry analyzer (Thermo Fisher) and the commercial detection kits listed in **Table S4**. Until measurement, samples were stored for up to 48 h at 4 °C. Daily calibration of the analyzer was performed according to the manufacturer's protocols.

Calculation of shear stress in the brain compartment

The average shear stress (τ) in the brain compartment can be calculated with the formula for the average wall shear stress in rectangular channels:

$$\tau = \frac{6 * \eta * \dot{V}}{h^2 * w}$$

In the presented system a dynamic viscosity (η) of 1 mPa*s, a height (h) of 0.1 mm, a compartment width (w) of 4.3 mm, a total flow rate ($\dot{V}$) of 16.9 µl/min and a brain compartment ratio of the total flow of 21.1% [2] are given.

LC/MS

All substance quantifications and metabolite identifications in this thesis were done by ultra-high-performance liquid chromatography high-resolution mass spectrometry (UHPLC-MS/HRMS (Q-Orbitrap)) performed by Pharmacelsus GmbH.

For metabolite identification, an identical instrument response was assumed for parent compounds and metabolites. Metabolite identification is based in accurate mass only. All peak areas were manually integrated.

Samples from BBB/brain and liver chip coculture were immediately frozen at -80 °C and sent frozen to Pharmacelsus GmbH for parent compound quantification and metabolite identification. AUC values of metabolites of propranolol and atenolol were measured in empty control chips and in coculture of the BBB/brain and the liver models with and without BMEC-like cells. Concentration ratios of the parent compound between the brain compartment and the circulation were calculated with quantified substance concentrations from individual CHIP4s with and without BMEC-like cells. Parent compound quantification was performed in three experiments. Metabolite identification was performed in one experiment.

CYP3A4 induction assay

The activity and induction of the cytochrome CYP3A4 by Rifampicin was measured with the P450 Glo CYP3A4 assay kit (Promega V9002). Twenty liver spheroids were collected per well of a 24-well ULA-plate in 500 µL liver maturation medium. CYP induction was initiated by addition of 10 µM rifampicin (Sigma-Aldrich) in liver maturation medium (composition found in the "Differentiation of hepatocyte-

like cells" section). As control conditions liver maturation medium with 0.04 % DMSO (solvent control) and pure liver maturation medium (negative control) were used. Treatment and control medium was exchanged every day for three days. After this induction phase, cell culture medium was removed and the spheroids washed with PBS+/+ to remove remaining rifampicin. Then, 3 µM CYP3A4/Luciferin-IPA in liver maturation medium was added to the spheroids and incubated overnight. To measure background luminescence by substances in the medium or degradation of the luminogenic CYP substrate, liver maturation medium with 3 µM CYP3A4/Luciferin-IPA was incubated without cells as a blank control. The next day 75 µL supernatant was transferred into a white 96-well plate with transparent bottom (Corning) and mixed with 75 µL luciferin detection reagent. The mixture was incubated for 20 min in the dark; the luminescence was then measured at a CLARIOstar plate reader (BMG labtech). All recorded luminescence intensities were corrected by the blank control without cells. Fold changes to the DMSO control were calculated by dividing the luminescence intensity of the treated samples by the luminescence intensity of the DMSO control.

Sodium-Fluorescein permeation

Sodium fluorescein (~0.4 kDa) was used for measuring permeability of the endothelial cell barrier before the chip experiment. The culture medium was removed and refilled in the lower Transwell culture chamber with 10 µM sodium fluorescein in BBB stage 2 medium. Empty 96-well Transwells with fibronectin / collagen IV coating were used in parallel as blank measurement. After application of the sodium-fluorescein solution, the models were incubated at an orbital shaker at 85 rpm for 60 min. After this permeation time, 50 µL were sampled on both sites of the membrane and transferred to a black 96-well plate. Pure BBB stage 2 medium and the application solution were used as controls. All samples were diluted 1:2 with $PBS^{-/-}$ before measurement. Both compounds were excited with a wavelength of 490/9 nm, and fluorescence emission was measured at 525/20 nm at the CLARIOstar plate reader (BMG Labtech). Fluorescence intensity of the medium blank was subtracted from all other readings before further analysis of the data.

The cell specific permeability coefficient PC_{cell} was calculated with the following set of equations, based on the clearance principle [3,4]. First, the substance clearing rate PS was calculated for empty (PS_{blank}) and BMEC-like cells seeded (PS_{all}) cell culture inserts:

$$PS_{all}\left[\frac{\mu l}{min}\right] = \frac{PA_{top} * V_{top}\ [\mu L]}{PA_{stock} * t[min]}$$

The incubation time of 60 min is t. To correct for the influence of the cell culture membrane the cell specific clearance rate PS_{cell} was calculated with following formula:

$$PS_{cell}\left[\frac{\mu l}{min}\right] = \frac{1}{\frac{1}{PS_{all}} - \frac{1}{PS_{blank}}}$$

To compare different membrane geometries the cell specific permeability coefficient PC_{cell} was calculated with following formula:

$$PC_{cell}\left[\frac{\mu m}{min}\right] = \frac{PS_{cell}\left[\frac{\mu l}{min}\right]}{A[mm^2]} * 1000$$

When no suitable blank value was available, the same formula was used to calculate the apparent permeability coefficient PC_{app} by using PS_{all} instead of PS_{cell} for calculation.

Supplemental References

1. Metsalu, T.; Vilo, J. ClustVis: A Web Tool for Visualizing Clustering of Multivariate Data Using Principal Component Analysis and Heatmap. *Nucleic Acids Res.* **2015**, *43*, W566–W570,

doi:10.1093/nar/gkv468.

2. Ramme, A.P.; Koenig, L.; Hasenberg, T.; Schwenk, C.; Magauer, C.; Faust, D.; Lorenz, A.K.; Krebs, A.-C.; Drewell, C.; Schirrmann, K.; et al. Autologous Induced Pluripotent Stem Cell-Derived Four-Organ-Chip. *Futur. Sci. OA* **2019**, *5*, FSO413, doi:10.2144/fsoa-2019-0065.

3. Neuhaus, W.; Plattner, V.E.; Wirth, M.; Germann, B.; Lachmann, B.; Gabor, F.; Noe, C.R. Validation of in Vitro Cell Culture Models of the Blood–Brain Barrier: Tightness Characterization of Two Promising Cell Lines. *J. Pharm. Sci.* **2008**, *97*, 5158–5175, doi:10.1002/jps.21371.

4. Novakova, I.; Subileau, E.A.; Toegel, S.; Gruber, D.; Lachmann, B.; Urban, E.; Chesne, C.; Noe, C.R.; Neuhaus, W. Transport Rankings of Non-Steroidal Antiinflammatory Drugs across Blood-Brain Barrier in Vitro Models. *PLoS One* **2014**, *9*, 1–14, doi:10.1371/journal.pone.0086806.
